# Genomic and Metabolic Features of the *Lactobacillus sakei* HRB10 Isolated from Traditional Dry Sausage in Northeast China Based on Whole Genome Sequencing Technology

**DOI:** 10.3390/foods15061089

**Published:** 2026-03-20

**Authors:** Qian Chen, Yunlong Bai, Yingying Fan, Jiasheng Lu, Yumeng Sui, Baohua Kong, Yingying Hu

**Affiliations:** 1 College of Food Science, Northeast Agricultural University, Harbin 150030, China; 2 School of Food and Biological Engineering, Hefei University of Technology, Hefei 230009, China

**Keywords:** genomic features, metabolic characteristics, traditional dry sausage, meat products, whole genome sequencing technology

## Abstract

This study aimed to analyze the whole genome sequencing of *Lactobacillus* (*Lb*) *sakei* HRB10, which was isolated from traditional dry sausage, to investigate its genetic traits and metabolic processes. The study revealed that the genome total length of *Lb sakei* HRB10 was 1987622 base pairs (bp), containing 1906 genes and a Genomic Component (GC) percentage of 41.11%. Database annotations indicate that the primary pathways in the genome of *Lb sakei* HRB10 are amino acid, fatty acid, and carbohydrate metabolisms. These pathways are crucial in forming the distinct flavor in dry sausage. There are many annotated genes encoding enzymes associated with amino acid and carbohydrate metabolisms, but there is a limited number of annotated genes encoding enzymes associated with fatty acid metabolism. Comparative genomics analysis results showed that the length of *Lb. sakei* HRB10 genomes were in the range of 1.93−2.07 Mb, and the GC content was 41.05−41.22%. The phylogenetic tree results and average nucleotide identity showed a very high homology between *Lb. sakei* HRB10, MFPB19, and TMW-1.3. This study provides knowledge to understand the formation mechanism of flavor formation by *Lb. sakei* HRB10 in dry sausages, thereby facilitating the identification of promising strains for application in meat fermentation.

## 1. Introduction

Dry sausage holds significant importance as a traditional fermented meat product in northeast China due to its unique texture and flavor [1]. Generally, dry sausages are prepared using pork meat and pork back fat, salt, nitrite spices, and sugar and undergo spontaneous fermentation in an open environment for a period of 10–15 days [2]. Dry sausages are often made following family customs and geographical factors specific to the region [3]. Fermentation is a complex and dynamic biochemical process affected mainly by the microbial composition and metabolic processes [4]. However, the microorganisms producing dry sausage exhibit significant differences due to the diverse conditions and methods, leading to product unpredictability and specific safety hazards [5]. Therefore, it is imperative to ensure consistent quality and safety for traditional dry sausages. Understanding the functional microorganisms involved in fermentation is essential for improving product safety, sensory quality, and the development of reliable starter cultures for the meat industry.

In this context, it was reported that inoculation of starter cultures was widely used to enhance traditional meat products’ quality and safety [6]. Similarly, Fuka et al. [7] found that the technological superiority of selected autochthonous starting cultures, which were specifically adapted for the meat matrix, exhibited technological superiority in terms of their resistance to high salt levels, growth capacity, resilience to low pH, and antimicrobial potential. Lactic acid bacteria (LAB) play a vital role in bio-preservation and safety assurance in the majority of fermented meat products [8]. LAB could have the ability to synthesize antimicrobial chemicals that can effectively control the growth of pathogenic and spoilage microorganisms [9]. Therefore, they are usually used as a starter culture in fermented meat products to drive fermentation and improve organoleptic properties, including texture, flavor, and overall appearance [10].

Our previous studies explored the relationship between volatile compounds’ production and bacterial communities in dry sausages. The results revealed that various LAB strains, including *Lactobacillus sakei*, *Lactobacillus plantarum*, *Weissella hellenica*, and *Lactococcus lactis*, could be linked to flavor development [11,12]. Additionally, our recent studies isolated 37 LAB strains and characterized their fermentation processes and flavor-enhancing abilities. Among these, *Lb. sakei* HRB10 might be considered a starter culture for improving fermented sausage’s flavor characteristics [2,13]. However, the mechanism by which *Lb. sakei* HRB10 contributes to flavor formation remains unclear, particularly at the genomic level.

Microorganisms’ physiological and metabolic functions can be investigated using whole genome sequencing [14]. In this context, Yao et al. [15] analyzed the whole genome of *Thermoactinomyces daqus* H-18. They discovered that it contains complete metabolic pathways for volatile compounds such as acetaldehyde, acetic acid, methyl butyrate, and methyl propionate. Similarly, Jacobs et al. [16] investigated the whole genome of *Bacillus cereus*. They observed that it has metabolic pathways for synthesizing phenylethanol, phenylacetic acid, benzoic acid, ethyl cinnamate, acetic acid, and lactic acid and has key enzymes for synthesizing various organic acids.

Although *Lb. sakei* is widely recognized as an important species in fermented meat products, increasing evidence indicates that different strains exhibit considerable variability in their genomic characteristics and metabolic capabilities, which may influence flavor formation and technological performance during fermentation [17]. However, the genetic basis underlying the flavor-related metabolic potential of specific *Lb. sakei* strains isolated from traditional dry sausages remains poorly understood. Thus, this research aimed to investigate the genetic characteristics and metabolic mechanisms of *Lb. sakei* HRB10 isolated from the traditional dry sausages. The pathways and enzymes related to carbohydrate, protein, and lipid metabolism were evaluated underlying fermented meat products’ flavor formation through genome sequencing analysis. Additionally, the comparative genome analysis of *Lb. sakei* regarding the genome evolution and relatedness were conducted. This study would enhance the understanding of the mechanism of flavor production by *Lb. sakei* HRB10, which will benefit the development of fermented meat products.

## 2. Materials and Methods

### 2.1. Strain Isolation and DNA Extraction

Our previous studies revealed that the *Lb. sakei* HRB10 strain was isolated from traditional dry sausage and had favorable technological properties and flavor-generating capability [2,13]. Using Man–Rogosa–Sharpe broth (Qingdao Hope Bio-Technology Co., Ltd., Qingdao, China), the *Lb. sakei* HRB10 strain was cultured until it reached the exponential growth phase and the DNA was extracted with Cetyltrimethylammonium Bromide method. The purity of extracted DNA was analyzed with the Implen NanoPhotometer^®^ spectrophotometer (Implen, Inc., Westlake Village, CA, USA). Genomic integrity and potential contamination were assessed by 1% agarose gel electrophoresis. All subsequent analyses in this study were conducted using this purified isolate to avoid potential interference from other microorganisms originally present in the sausage microbiota.

### 2.2. Whole Genome Sequencing and Bioinformatics Analysis

#### 2.2.1. PacBio Sequencing

Covaris G-tubes (Woburn, MA, USA) were used for fragmentation of the genetic DNA. End-repairing of qualified DNA fragments was used to make SMRTbell DNA template libraries with fragment sizes of more than 10 kb. The size of DNA fragments was selected using a blue pippin system (PacBio, Menlo Park, CA, USA). Qubit^®^ 2.0 Fluorometer (Life Technologies, Carlsbad, CA, USA) was utilized to detect the library quality. Agilent Bioanalyzer 2100 (Santa Clara, CA, USA) was used to estimate average fragment size, and SMRT sequencing was conducted using Pacific Biosciences Sequel (Menlo Park, CA, USA).

#### 2.2.2. Illumina Sequencing

The genomic DNA that met the specified criteria was randomly sonicated. After that, using NEBNext^®^ MLtra™ (DNA Library Prep Kit for Illumina, NEB, San Diego, CA, USA), the sonicated DNA was end-repaired, A-tailed, and adaptor-ligated. The PCR technique was used to enrich DNA fragments of 300–400 bp in length. Finally, the AMPure XP device from Beckman Coulter in Brea (Beckman Coulter, Brea, CA, USA) was used for purification of the PCR products. A size distribution analysis was conducted on the subsequent libraries using the 2100 Bioanalyzer from Agilent in Santa Clara, CA, USA. The quantification was performed using real-time PCR. The genome underwent sequencing utilizing the Illumina Novaseq 6000 sequencer equipped with the PE 150 pair-end technology capability.

#### 2.2.3. Assembly and Annotation

De novo assembly was performed using Falcon (version 0.3.0) on long reads acquired from single-molecule radiofrequency (SMRT) sequencing runs. Then, using FASTP (version 0.20.0), the Illumina platform raw data underwent filtration using FASTP (version 0.20.0). The following criteria was used to filter data: (1) eliminating reads with 10% or more unidentified nucleotides (N); (2) eliminating reads with 50% or more bases with phred quality scores of 20 or lower; (3) eliminating reads aligned to the barcode adaptor. To optimize the assembly quality and determine the final genome sequences, the clean reads acquired after filtering were utilized to correct the genome sequences using Pilon (version 1.23) [18].

The NCBI prokaryotic genome annotation or Prokka (version 1.11) approaches were used for predictions of protein-coding genes. The prediction of noncoding RNAs, specifically rRNAs, was performed with rRNAmmer (version 1.2). tRNAs were identified using tRNAscan (version 1.3.1), while sRNAs were predicted using cmscan (version 1.1.2). Formal annotation of the assembled sequences was conducted by referring databases, including Gene Ontology (GO), Kyoto Encyclopedia of Genes and Genomes (KEGG), Carbohydrate-Active enzymes (CAZy), and Cluster of Orthologous Groups of proteins (COG).

### 2.3. Comparative Genomics Analysis

#### 2.3.1. Core-Pan Genome Analysis

The genomic sequence of *Lb. sakei* HRB10 was uploaded to the two GenBank (accession number CP101642). Comparative genome analysis utilized five genome sequences acquired from the NCBI microbial genome database, such as *Lb. sakei* WiKim0063 (Accession SAMN07454401), *Lb. sakei* TMW-1.1239 (Accession SAMN04606860), *Lb. sakei* TMW-1.3 (Accession SAMN04606855), *Lb. sakei* ZFM225 (Accession SAMN07311129), and *Lb. sakei* MFPB19 (Accession SAMEA104285093).

The protein sequences of all strains were subjected to CD-HIT-based clustering analysis for the construction of the core-pan genome. The pan genome encompasses all genes found in the population of closely related species. The sequences inside each strain in the pan genome are defined as the core genome [19].

#### 2.3.2. Phylogenetic Tree Construction

Following the method described by Nguyen et al. [20], a phylogenetic tree was generated using the maximum likelihood approach, including the core gene sequences of the genomes of 6 *Lb. sakei*. The phylogenetic tree to examine the evolutionary relationships among *Lb. sakei* strains was built with the iqtree (version 1.6.3) module.

#### 2.3.3. Average Nucleotide Identity (ANI) Analysis

ANI is an indicator for comparing the genetic relationship of the two genomes at the nucleotide level. ANI was calculated using pyani (version 0.2.7) for two genomic comparisons between 6 *Lb. sakei* strains [21].

## 3. Results

### 3.1. General Genome Features

**Figure 1:**
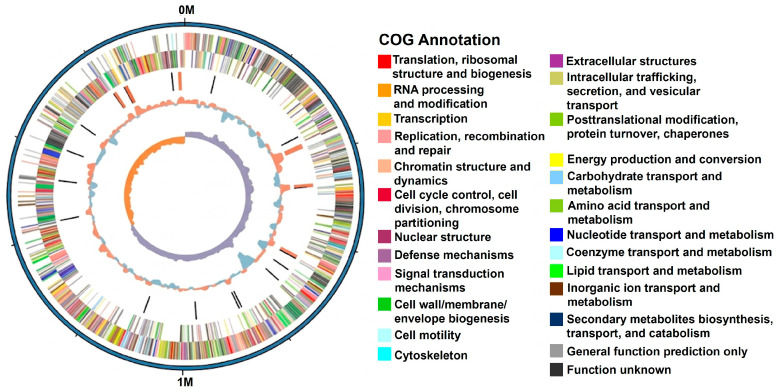
Map of the *Lb. sakei* HRB10 chromosome. The outer circle represents the genome size in kb. The second and third circles represent the predicted coding sequences (CDSs), and the different colors indicate the different clusters of orthologous groups of proteins (COGs). Moving inward, the fourth circle represents the rRNA and tRNA clusters. The fifth circle represents the GC content with red and blue color, and the most inner circle represents GC-skew values.

The results revealed that the whole genome consisted of a 1,987,622 bp (GC content of 41.11%) circular chromosome (GenBank accession number CP101642). The chromosome was predicted to encode 1906 genes based on functional annotation of NR, KEGG, GO, and COG databases. RNAmmer 1.2 Server predicted the presence of 65 tRNA genes and 21 rRNA genes (7 16S rRNA genes, 7 23S rRNA genes, and 7 5S rRNA genes) as shown in Table 1. In order to obtain comprehensive and macro-level views of the gene distribution characteristics on the chromosome of *Lb. sakei* HRB10, the circular genome maps were generated using the CG View Server (Figure 1). These genomic characteristics are comparable to those reported for other *Lb. sakei* strains, which typically possess genomes of approximately 1.8–2.1 Mb with similar GC content, suggesting a relatively conserved genomic structure within this species [22].

### 3.2. Annotation of Functional Genes in the Genome of Lb. sakei HRB10

#### 3.2.1. COG Database Annotation

**Figure 2:**
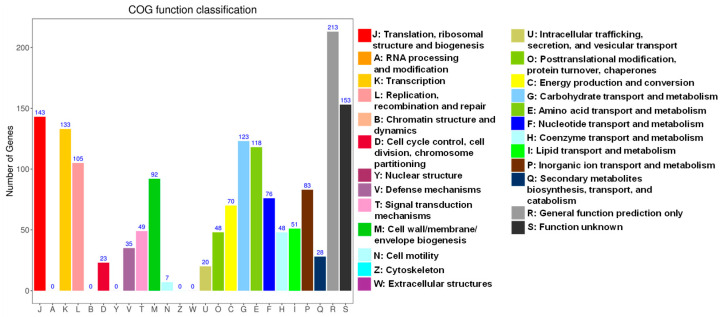
Gene function annotation of *Lb. sakei* HRB10 genome based on the COG database.

Figure 2 presents the COG annotation of *Lb. sakei* HRB10 genome. Annotations were made to a total of 1395 genes out of the 1906 identified coding genes. A large proportion of genes were involved in translation, ribosomal structure and biogenesis, transcription, recombination and repair, and replication, as well as amino acid and carbohydrate transport and metabolism. In particular, genes related to amino acid transport and metabolism and carbohydrate transport and metabolism accounted for a considerable proportion of the annotated functions. These functional categories suggest that *Lb. sakei* HRB10 possesses active metabolic capabilities and strong growth potential. The dominance of genes involved in carbohydrate and amino acid metabolism is consistent with previous genomic studies of *Lb. sakei*, which have shown that these metabolic features contribute to the adaptation of the species to meat environments and support microbial growth during sausage fermentation [23].

#### 3.2.2. KEGG Database Annotation

**Figure 3:**
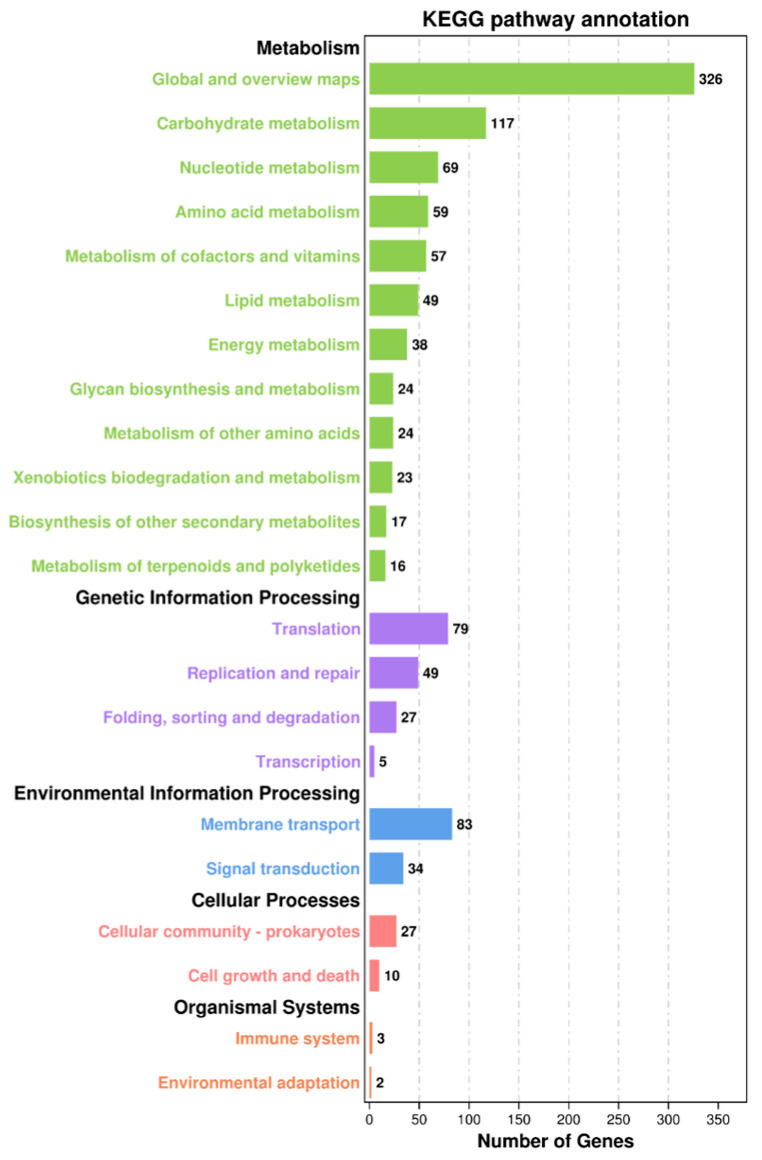
Gene function annotation of *Lb. sakei* HRB10 genome based on the KEGG database.

As shown in Figure 3, annotation of the 1100 genes was performed using the KEGG database. The KEGG database categorizes metabolic pathways into five groups based on their function. In the corresponding categories of metabolism, genetic information processing, environmental information processing, cellular processes, and organismal systems, the number of genes was 819, 160, 117, 37, and 5. Furthermore, the genes implicated in metabolism constituted the largest percentage. Within the category of metabolism genes, the genes associated with global and overview maps had the greatest prevalence. This was followed by genes related to carbohydrate metabolism, nucleotide metabolism, amino acid metabolism, metabolism of cofactors and vitamins, and lipid metabolism. These metabolic pathways are considered important for the formation of flavor-related metabolites in fermented meat products, as carbohydrate and amino acid metabolism can generate organic acids, aldehydes, alcohols, and other volatile compounds contributing to the characteristic aroma of fermented sausages [23].

#### 3.2.3. GO Database Annotation

**Figure 4:**
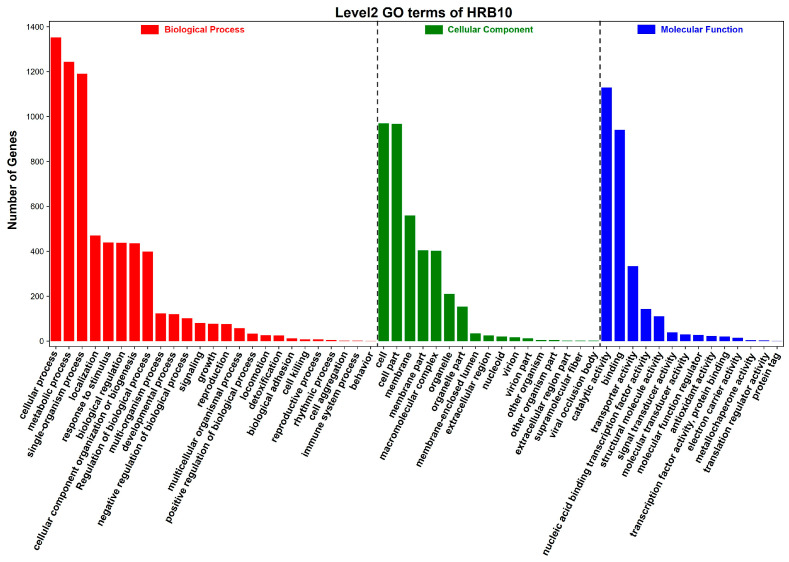
Gene function annotation of *Lb. sakei* HRB10 genome based on the GO database.

Overall, 1613 genes were annotated using GO categorization (Figure 4). The biological process category has the highest number of GO keywords and gene count (2496), followed by molecular function (1518 genes) and cellular component (1100 genes). In the biological process, the most significant pathways were cellular process (1352 genes), metabolic process (1244 genes), and single-organism process (1191 genes). Catalytic activity (1129 genes) and binding (941 genes) represented the significant pathways in the molecular function, while cell (970 genes) and cell part (967 genes) were the most significant pathways in the cellular component.

#### 3.2.4. Analyses of CAZymes

The numerous kinds CAZymes capable of assembling, modifying, and degrading oligo- and polysaccharides are widely found in LAB. According to the findings of Lombard et al. [24], the carbohydrate-active enzymes (CAZy) database is utilized for the analysis of genetic, structural, and biochemical data pertaining to CAZy enzymes specifically involved in the degradation, modification, or synthesis of glycosidic linkages. As shown in Table 1, GHs and GTs had the highest number of CAZy genes in *Lb. sakei* HRB10 genome. Altogether, 123 genes encoding glycosyl transferases (GTs) were allocated to 20 GT families, such as GT0, GT1, GT13, GT2, GT26, GT27, GT28, GT29, GT30, and GT32. GT is associated with the production of oligosaccharides and polysaccharides, which possess immunomodulatory, tumor suppressive, antioxidant, and gut microbial control properties [25,26]. Moreover, GH enzymes possess the capacity to break down intricate carbohydrates, which include crucial enzymes involved in carbohydrate metabolism [27,28]. A total of 69 genes were annotated into 38 glycoside hydrolases (GHs), such as GH13, GH133, GH135, GH16, GH19, GH2, GH23, and GH25. These GHs can utilize a wide variety of substrates such as starch/glycogen, peptidoglycan, sucrose/fructans, plant cell wall carbohydrates, animal carbohydrates, and other carbohydrates. Furthermore, the prediction indicated the presence of one gene for polysaccharide lyases (PL), 14 genes for carbohydrate esterases (CEs), three genes for auxiliary activities (AAs), and 38 genes for carbohydrate-binding modules (CBMs).

### 3.3. Carbohydrate Metabolic Pathways of Lb. sakei HRB10

Microbial growth and the production of flavor compounds were both based on carbohydrate metabolism [29]. As shown in Table S1, around 18 sugar phosphotransferase systems (PTS) were identified in the genome of *Lb. sakei* HRB10. These PTS catalyze the phosphorylation of incoming sugars and facilitate their transport into the cell [30]. According to the annotation, these PTS could transport various carbohydrates, including the sucrose, N-acetylgalactosamine, galactosamine, cellobiose, β-glucoside, glucitol/sorbitol, fructose, and mannose. These PTS transport phosphorylated sugar derivatives that assist the central carbon metabolism. Study of the annotated whole genome of *Lb. sakei* HRB10 verified the existence of the genes that encode all the enzymes necessary for the glycolytic pathway, which converts glucose into pyruvate (Figure S1; Table S2).

Pyruvate, which is generated by glycolysis, is a crucial intermediary in the process of flavor development during the fermentation of fermented sausage [31]. As shown in Figure S2 and Table S3, *Lb. sakei* HRB10 genome contains genes coding for L-lactate dehydrogenase (NON14_03130; NON14_09225) that can directly convert pyruvate to lactate. Meanwhile, the pyruvate also can be converted to the acetate via the genes encoding pyruvate oxidase (NON14_01980) and acylphosphatase (NON14_04405) in *Lb. sakei* HRB10 genome. These organic acids generated from carbohydrate metabolism during fermentation can provide sourness and lower pH, thereby improving the flavor and safety of fermented sausage [32]. In addition, the pyruvate also can be converted the acetyl-CoA via the pyruvate dehydrogenase E1 component alpha subunit (NON14_05855), pyruvate dehydrogenase E1 component beta subunit (NON14_05860), pyruvate dehydrogenase E2 component (NON14_05865), and dihydrolipoamide dehydrogenase (NON14_05870). The acetyl-CoA is the starting intermediate of the tricarboxylic acid (TCA) cycle and is the important precursor of amino acids and fatty acids, contributing to further generate flavors (e.g., organic acids and ethanol) [33]. Three acetyl-CoA conversion pathways were detected in the *Lb. sakei* HRB10 genome. The first pathway was that acetyl-CoA could be further metabolized by genes encoding acetyl-CoA C-acetyltransferase (NON14_03875) to acetoacetyl-CoA, which could be involved in butanoate metabolism. The second pathway was that acetyl-CoA could be further metabolized by genes encoding *acc* (NON14_07115, NON14_07120, NON14_07125, NON14_07135) to malonyl-CoA, which could be involved in and fatty acid biosynthesis. The third pathway was that acetyl-CoA could be converted to the acetaldehyde via the gene encoding acetaldehyde dehydrogenase/alcohol dehydrogenase (NON14_09700) in the *Lb. sakei* HRB10 genome. Furthermore, the fourth pathway was that acetyl-CoA could be involved in the TCA cycle to facilitate the conversion of citric acid to lactic acid, acetic acid, acetoin, and other substances [3]. It was worth noting that ester compounds presenting fruity, sweet, and floral aromas were frequently detected in fermented sausage, which could be formed by the esterification of organic acids and alcohols generated during fermentation [34].

### 3.4. Proteolytic System and Amino Acids Metabolism of Lb. sakei HRB10

There were oligopeptides transport system (Opp) composed of oligopeptide-binding protein (OppA), two integral membrane proteins (OppB and OppC), and two nucleotidebinding proteins (OppD and OppF) in *Lb. sakei* HRB10 genome (Table S4), which have the ability to transport di-, tripeptides, and oligopeptides into the cell [35,36,37]. These peptides can be degraded by a variety of peptidases (e.g., aminopeptidases, oligo-/di-/tri-peptidases, proline peptidase, carboxypeptidases, endopeptidase, and uncharacterized peptidase) in *Lb. sakei* HRB10 genome (Table S5), suggesting that it has a relatively complete peptidase system to produce amino acids [38].

Amino acids are one of the nitrogen sources for the growth and metabolism of microorganisms, and they are important precursors of higher alcohols, esters, and other flavor compounds [39], such as the branched-chain amino acids (valine, leucine, and isoleucine), the aromatic amino acids (tyrosine, tryptophan, and phenylalanine), and the sulfur-containing amino acids (methionine and cysteine) [40]. The relevant pathways of amino acid metabolism involve dehydrogenation, decarboxylation, transamination, and oxidation reactions, which are related to the transaminase, dehydrogenase, decarboxylase, oxidase, lyase, etc. [41]. It is well known that transaminases catalyze the transfer of amino groups from amino donors to amino acceptor compounds [42]. Amino acids are catalyzed by transaminase and decarboxylase to sequentially generate α-keto acids and aldehydes. However, there are only transaminase *glmS* (NON14_04530) and aspartate 4-decarboxylase *asdA* (NON14_01735) in *Lb. sakei* HRB10 genome shown in Table S6. Then, the α-keto acids and aldehydes could be further converted into the α-hydroxy acids and alcohols/carboxylic acids under the catalysis of alcohol dehydrogenase, respectively [43]. There are many dehydrogenase in *Lb. sakei* HRB10 genome, such as alcohol dehydrogenase *yiaY* (NON14_01520), alcohol dehydrogenase *adhP* (NON14_02650), alcohol dehydrogenase *E1.1.1.1* (NON14_06630), alcohol dehydrogenase *adhE* (NON14_09700), L-lactate dehydrogenase *LDH* (NON14_03130, NON14_09225), dihydrolipoamide dehydrogenase *DLD* (NON14_05870), shikimate dehydrogenase aroE (NON14_06785), aryl-alcohol dehydrogenase *E1.1.1.90* (NON14_08700). Collectively, in the current study, *Lb. sakei* HRB10 harbored many genes for enzymes involved in amino acid metabolism, but it still needs to cooperate with other microorganisms on amino acid metabolism to promote the flavor formation in fermented sausage.

In addition to the metabolic pathways described above, the genomic features of *Lb. sakei* HRB10 also suggest its potential contribution to flavor development during fermented sausage maturation. Carbohydrate metabolism provides key intermediates such as pyruvate and acetyl-CoA, which can be further converted into organic acids, alcohols, and ester precursors that contribute to the typical sour and fermented aroma of meat products [44]. Meanwhile, the presence of a relatively complete proteolytic system and multiple peptidases enable the release of amino acids from peptides, which can act as important precursors of volatile flavor compounds such as aldehydes, alcohols, and acids through transamination and dehydrogenation reactions [45]. In addition, alcohol dehydrogenases and aldehyde dehydrogenases identified in the *Lb. sakei* HRB10 genome may facilitate the conversion of aldehydes into alcohols and other aroma-active compounds. Previous studies have reported that these metabolic activities of *Lb. sakei* strains are closely associated to the formation of characteristic flavor compounds in fermented meat products. Therefore, the genomic characteristics of HRB10 indicate that this strain may contribute to flavor development and sensory quality during sausage fermentation, which may provide potential advantages for its application as a starter culture in fermented meat products.

### 3.5. Lipid Degradation and Fatty Acid Metabolic Pathways of Lb. sakei HRB10

The fatty acid biosynthesis pathway was the principal pathway of aroma compounds formation. As shown in Table S7 and Figure S3, there are all genes identified encoding for enzymes related to fatty acid biosynthesis in the *Lb. sakei* HRB10genome. Acetyl-CoA is catalyzed by acetyl-CoA carboxylase *acc* (NON14_07115, NON14_07120, NON14_07125, NON14_07135) to transfer the carboxyl group of bicarbonate to acetyl group to obtain malonyl-CoA with energy produced by ATP hydrolysis. Then, the malonyl group is transferred to the acyl carrier protein to act as a carbon donor for fatty acid synthesis catalyzed by S-malonyl transferase *fabD* (NON14_07150) [39]. The reductase *fabG* (NON14_07145, NON14_09010, NON14_09175) is also a key enzyme that can reduce 3-carbonyl oxygen at the acyl end to -OH, and then the hydroxyl is removed to obtain the fatty acyl protein complex catalyzed by the dehydratase *fabZ* (NON14_07130, NON14_07165) and the reductase *fabI* (NON14_07110). Thioesterase *MCH* (NON14_09870) is also a key enzyme that can break the carbon–sulfur bond and link the hydroxyl group to form fatty acid and free acyl carrier protein. The fatty acid can further act as precursors to promote the formation of alcohols [46]. However, there are only a few genes identified encoding for enzymes related to fatty acid degradation in the *Lb. sakei* HRB10 genome (Table S8). The aldehyde dehydrogenase *adhE* (NON14_09700) encoded by *Lb. sakei* HRB10 can reduce the carboxyl group of fatty acids to the aldehyde group, and then the aldehyde group was reduced to the hydroxyl group by alcohol dehydrogenase *yiaY* (NON14_01520) to obtain alcohol [47]. Both reaction processes require the participation of NADH and reduced protons. On the whole, the lipid metabolism ability of *Lb. sakei* HRB10 is weak, even though it encodes a relatively complete fatty acid synthesis pathway.

### 3.6. Comparative Genomics Analysis

#### 3.6.1. General Genomic Characteristics of the *Lb. sakei*

As shown in Table 2, genomes in the *Lb. sakei* had a low GC content ranging from 41.05% to 41.22%. The genome sizes of the 6 *Lb. sakei* strains ranged from 1.93 Mb to 2.07 Mb, with 1679 to 1964 predicted coding-genes. These differences in genome size may indicate that the evolution of *Lb. sakei* is coupled with different levels of horizontal gene transfer, including gene insertion and deletion [48].

#### 3.6.2. Core-Pan Genome Analysis

**Figure 5:**
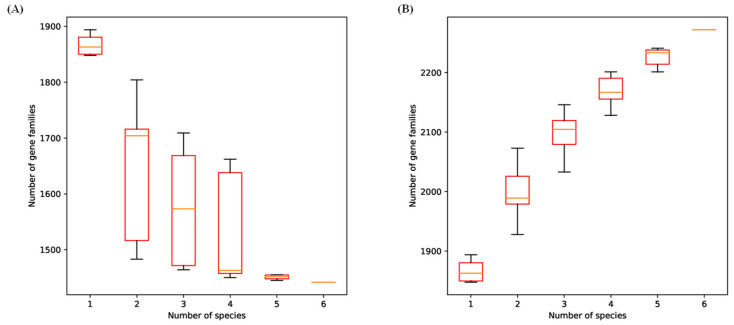
Core gene (**A**) and pan gene family (**B**) curve of *Lb. sakei*. Boxes represent the interquartile range (25th–75th percentiles) with the median indicated by the horizontal line. Whiskers indicate values within 1.5 × IQR, and points outside the whiskers represent outliers.

The pan gene and core gene sets of *Lb. sakei* were constructed to reveal the genomic differences among different *Lb. sakei* strains. Pan genome is a powerful concept that can be used to effectively represent the genomic features of a bacterial lineage, and its analysis can provide insights into the genome dynamics and evolution of the lineage. Core genome is a gene family shared by all strains, which are generally related to biological functions and main phenotypic characteristics of species, reflecting the stability of species. A pair of genes can be classified into the same gene family if their amino acid sequence similarity is more than 95% [49]. As shown in Figure 5, 1442 core genes and 2272 pan genes were identified in *Lb. sakei* genomes. With the addition of new strains, the number of core genomes in the gene accumulation curve was decreasing, while the number of pan genomes was gradually increasing, and both of them were gradually leveling off.

#### 3.6.3. Phylogenetic Tree Analysis

**Figure 6:**
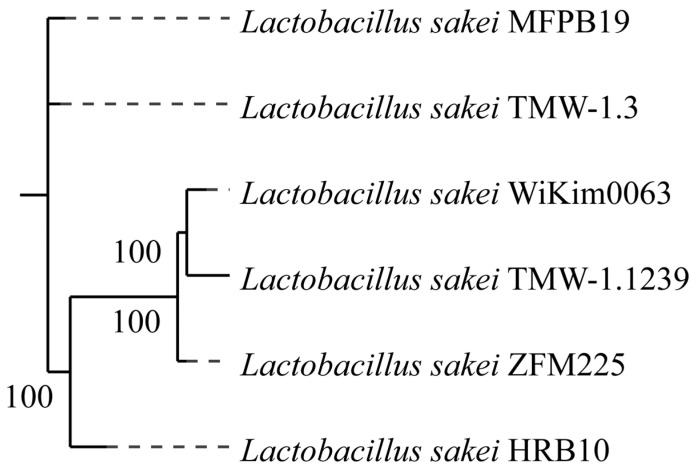
Phylogenetic tree of *Lb. sakei* based on core gene sequences. **Solid lines** represent branches with high support (bootstrap ≥ 70%), while **dashed lines** indicate branches with low support (bootstrap < 70%).

Genetic evolution relationship and population structure among strains can be reflected by phylogenetic tree [48]. The 1442 core genes identified by Roary software were used to construct phylogenetic trees to evaluate the intraspecific genetic evolution relationship between *Lb. sakei* HRB10 and the other five *Lb. sakei* strains. As shown in Figure 6, the phylogenetic tree was divided into two branches due to genetic diversity in the evolution process. *Lb. sakei* MFPB19, TMW-1.3, and HRB10 were close in genetic distance, which may be due to the fact that these strains are all isolated from meat products. *Lb. sakei* WiKim0063, TMW-1.1239, and ZFM225 that are isolated from pickles, sour dough, and milk, respectively, were close in genetic distance. Overall, the phylogenetic relationship of these strains may be related to the isolation source. The differences of core genes may be due to the differences in adaptability of strains in different environments, resulting in a certain isolation source specificity [50].

#### 3.6.4. ANI Analysis

**Figure 7:**
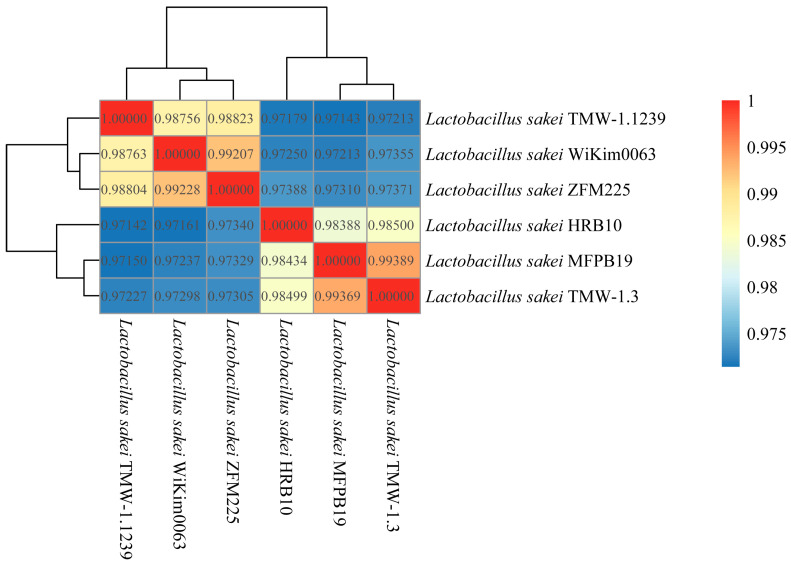
Clustering heatmap of ANI of *Lb. sakei*.

ANI is used to identify the genetic relationship of strains by comparing the homologous sequences of genome. It is generally considered that the ANI value greater than 95% is the same species [51]. As shown in Figure 7, the ANI values of *Lb. sakei* were greater than 97% when compared to each other, indicating that the strains used in the study belong to the same species. In addition, the ANI values between *Lb. sakei* HRB10 and MFPB19 or TMW-1.3 was higher than those of the other strains, indicating that the genomes of these three strains have very high homology, which was verified by the above results of the phylogenetic tree.

## 4. Conclusions

This study provides a comprehensive perspective of the genomic and metabolic features of *Lb. sakei* isolated from the traditional dry sausage. According to the annotation of databases, carbohydrate metabolism, amino acid metabolism, and fatty acid metabolism were the main pathways in *Lb. sakei* HRB10 genome, which are also important for the development of the characteristic flavor in dry fermented sausage. In terms of metabolic pathways in the *Lb sakei* HRB10 genome, there are many annotated genes encoding enzymes related to carbohydrate metabolism and amino acid metabolism, but few annotated genes encoding enzymes related to fatty acid metabolism. Comparative genomics analysis showed that each of the investigated *Lb sakei* strains harbors a high unique genetic diversity. Furthermore, more studies regarding the dynamic expression and regulation of enzyme-coding genes in the strain are also necessary. The outcome provides knowledge to understand the role of this strain in the flavor formation of dry sausage, which could contribute to the development of potential strains for utilization in other food fermentation and better flavor production.

## Figures and Tables

**Table 1:** General genome features and CAZymes-encoding genes of *Lb. sakei* HRB10.

	Strain	*Lb. sakei* HRB10
**General genome features**	Size (bp)	1987622
	GC content (%)	41.11
	Protein-coding genes	1906
	tRNA	65
	5S_rRNA	7
	16S_rRNA	7
	23S_rRNA	7
**CAZymes-encoding genes**	Glycosyl transferases	123
	Glycoside hydrolases	69
	Polysaccharide lyases	1
	Carbohydrate esterases	14
	Auxiliary activities	3
	Carbohydrate-binding modules	38

**Table 2:** Genomic information of *Lb. sakei* for the comparative genome analysis.

Strain	Source	Size (Mb)	GC Content (%)	Gene Number	CDS Number	GenBank Accession Number
*Lb. sakei* WiKim0063	Kimchi	2.07	41.17	2079	1964	SAMN07454401
*Lb. sakei* ZFM225	Raw cow milk	2.01	41.22	2039	1924	SAMN07311129
*Lb. sakei* MFPB19	Beef carpaccio	2.06	41.05	2098	1958	SAMEA104285093
*Lb. sakei* TMW-1.1239	Sourdough	1.98	41.20	1981	1873	SAMN04606860
*Lb. sakei* TMW-1.3	Sausage	1.93	41.12	1950	1679	SAMN04606855
*Lb. sakei* HRB10	Dry sausage	1.99	41.11	1992	1906	CP101642

## Data Availability

The original contributions presented in this study are included in the article/Supplementary Materials. Further inquiries can be directed to the corresponding author.

## Supplementary Materials

The following supporting information can be downloaded at: https://www.mdpi.com/article/10.3390/foods15061089/s1, Figure S1: Glycolytic pathway of *Lb. sakei* HRB10; Figure S2: Pyruvate metabolic pathway of *Lb. sakei* HRB10; Figure S3: Fatty acid biosynthesis pathway of *Lb. sakei* HRB10. Table S1: Phosphotransferase system associated genes involved in *Lb. sakei* HRB10; Table S2: Glycolytic pathway associated genes involved in *Lb. sakei* HRB10; Table S3: Pyruvate metabolic pathway associated genes involved in *Lb. sakei* HRB10; Table S4: ABC transporters associated genes involved in *Lb. sakei* HRB10 genome; Table S5: Proteases and peptidases associated genes involved in *Lb. sakei* HRB10; Table S6: Amino acid metabolism associated genes involved in *Lb. sakei* HRB10; Table S7: Fatty acid biosynthesis pathway-associated genes involved in *Lb. sakei* HRB10; Table S8: Fatty acid degradation pathway-associated genes involved in *Lb. sakei* HRB10.
