# Supplementary material for: Genomic and Metabolic Features of the Lactobacillus sakei HRB10 Isolated from Traditional Dry Sausage in Northeast China Based on Whole Genome Sequencing Technology
Source: Foods. 2026 Mar 20;15(6):1089. doi: 10.3390/foods15061089 (PMC13025413; doi:10.3390/foods15061089)
Supplement: Supplementary file 1 [file foods-15-01089-s001.zip › foods-4178572-supplementary.pdf]

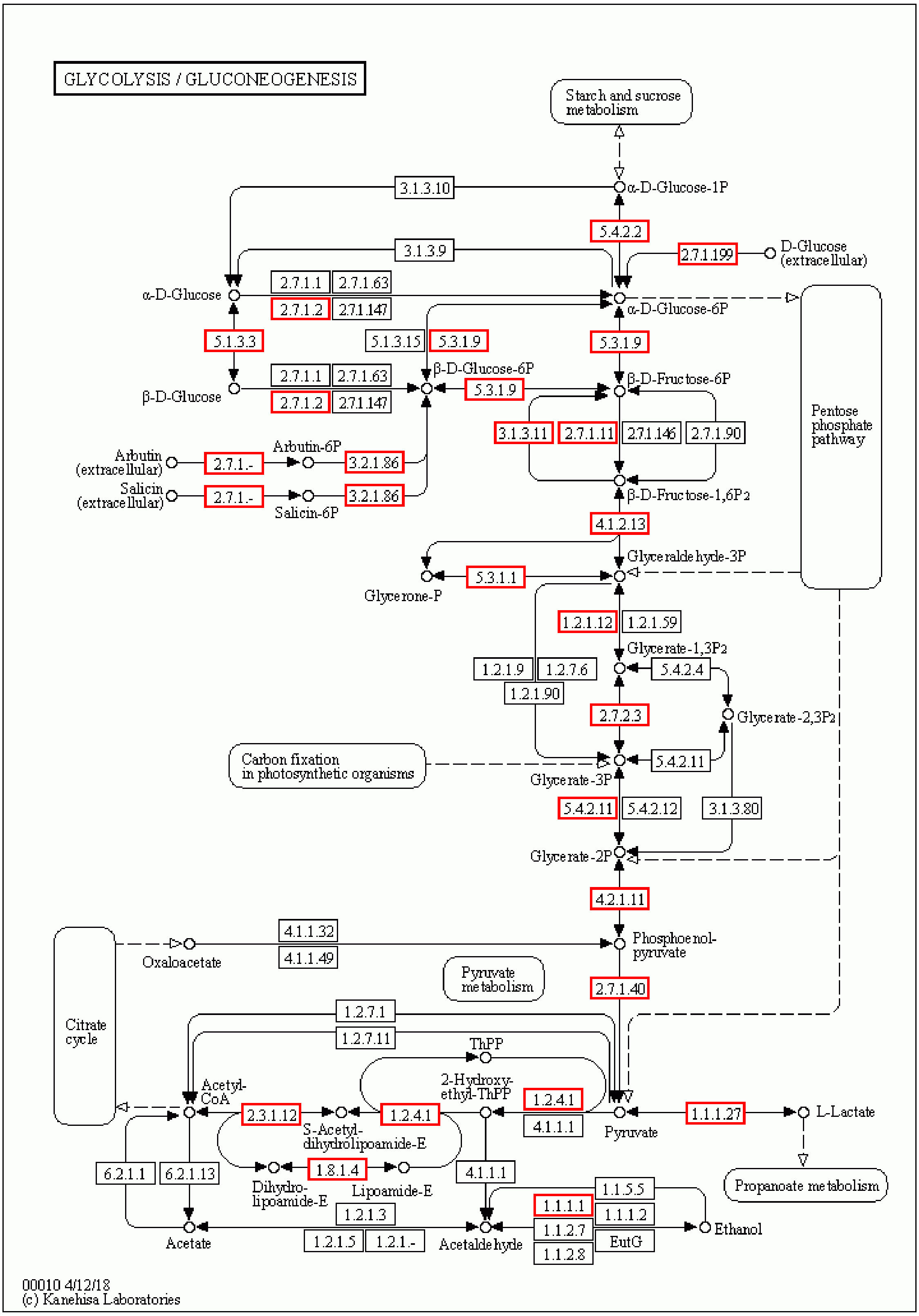

Figure. S1. Glycolytic pathway of *Lb. sakei* HRB10.

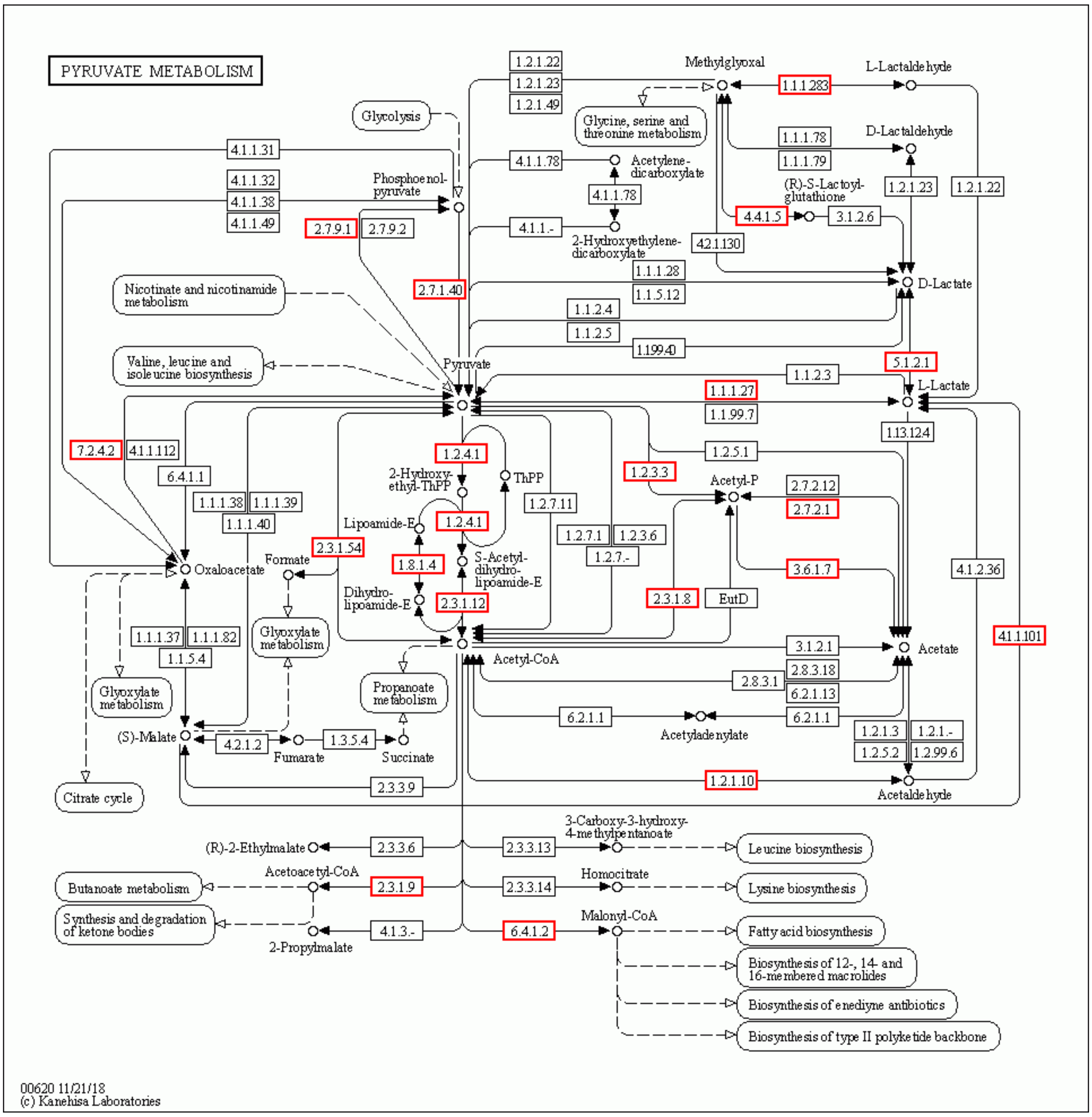

Figure. S2. Pyruvate metabolic pathway of *Lb. sakei* HRB10.

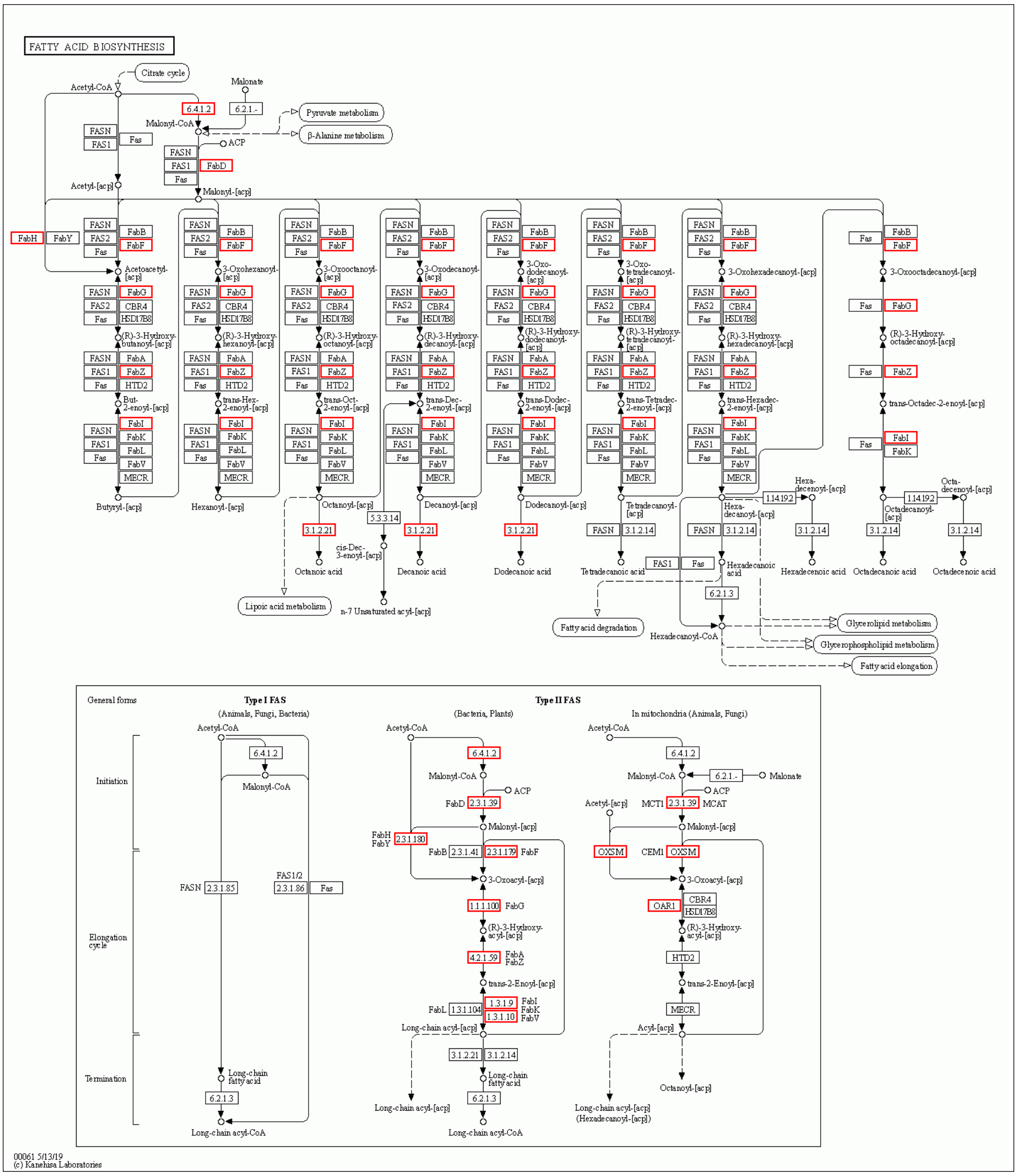

Figure. S3. Fatty acid biosynthesis pathway of *Lb. sakei* HRB10.

Table S1. Phosphotransferase system-associated genes involved in *Lb. sakei* HRB10.

Locus tag	KO	Gene_name	EC	Definition
NON14_00265	K10984	agaB	EC:2.7.1.-	galactosamine PTS system EIIB component
NON14_00270	K10985	agaC		galactosamine PTS system EIIC component
NON14_00275	K10986	agaD		galactosamine PTS system EIID component
NON14_00280	K02744	agaF	EC:2.7.1.-	N-acetylgalactosamine PTS system EIIA component
NON14_00915	K02761	celB		cellobiose PTS system EIIC component
NON14_02155	K02810	scrA	EC:2.7.1.211	sucrose PTS system EIIBCA or EIIBC component
NON14_03500	K02761	celB		cellobiose PTS system EIIC component
NON14_03545	K02759	celC	EC:2.7.1.196 2.7.1.205	cellobiose PTS system EIIA component
NON14_03985	K02784	ptsH		phosphocarrier protein HPr
NON14_03990	K08483	ptsI	EC:2.7.3.9	phosphoenolpyruvate-protein phosphotransferase (PTS system enzyme I)
NON14_04015	K02761	celB		cellobiose PTS system EIIC component
NON14_05285	K02757	bglF	EC:2.7.1.-	beta-glucoside PTS system EIICBA component
NON14_05295	K02777	crr	EC:2.7.1.-	sugar PTS system EIIA component
NON14_06030	K02770	fruA	EC:2.7.1.202	fructose PTS system EIIBC or EIIC component
NON14_06035	K00882	fruK	EC:2.7.1.56	1-phosphofructokinase
NON14_09065	K02781	srlB	EC:2.7.1.198	glucitol/sorbitol PTS system EIIA component
NON14_09190	K02796	manZ		mannose PTS system EIID component
NON14_09195	K02795	manY		mannose PTS system EIIC component
NON14_09200	K02794	manX	EC:2.7.1.191	mannose PTS system EIIAB component
NON14_09835	K02760	celA	EC:2.7.1.196 2.7.1.205	cellobiose PTS system EIIB component

Table S2. Glycolytic pathway-associated genes involved in *Lb. sakei* HRB10.

**Locus tag**	**KO**	**Gene_name**	**EC**	**Definition**
NON14_00510	K01834	PGAM	EC:5.4.2.11	2,3-bisphosphoglycerate-dependent phosphoglycerate mutase
NON14_00920	K01223	E3.2.1.86B	EC:3.2.1.86	6-phospho-beta-glucosidase
NON14_01040	K01834	PGAM	EC:5.4.2.11	2,3-bisphosphoglycerate-dependent phosphoglycerate mutase
NON14_01520	K13954	yiaY	EC:1.1.1.1	alcohol dehydrogenase
NON14_02285	K04041	fbp3	EC:3.1.3.11	fructose-1,6-bisphosphatase III
NON14_02650	K13953	adhP	EC:1.1.1.1	alcohol dehydrogenase, propanol-preferring
NON14_03130	K00016	LDH	EC:1.1.1.27	L-lactate dehydrogenase
NON14_03555	K01223	E3.2.1.86B	EC:3.2.1.86	6-phospho-beta-glucosidase
NON14_03575	K01624	FBA	EC:4.1.2.13	fructose-bisphosphate aldolase, class II
NON14_04660	K00845	glk	EC:2.7.1.2	glucokinase
NON14_05295	K02777	crr	EC:2.7.1.-	sugar PTS system EIIA component
NON14_05375	K01810	GPI	EC:5.3.1.9	glucose-6-phosphate isomerase
NON14_05855	K00161	PDHA	EC:1.2.4.1	pyruvate dehydrogenase E1 component alpha subunit
NON14_05860	K00162	PDHB	EC:1.2.4.1	pyruvate dehydrogenase E1 component beta subunit
NON14_05865	K00627	DLAT	EC:2.3.1.12	pyruvate dehydrogenase E2 component (dihydrolipoamide acetyltransferase)
NON14_05870	K00382	DLD	EC:1.8.1.4	dihydrolipoamide dehydrogenase
NON14_06110	K00850	pfkA	EC:2.7.1.11	6-phosphofructokinase 1
NON14_06115	K00873	PK	EC:2.7.1.40	pyruvate kinase
NON14_06630	K00001	E1.1.1.1	EC:1.1.1.1	alcohol dehydrogenase
NON14_07385	K01785	galM	EC:5.1.3.3	aldose 1-epimerase
NON14_08450	K01689	ENO	EC:4.2.1.11	enolase
NON14_08455	K01803	TPI	EC:5.3.1.1	triosephosphate isomerase (TIM)
NON14_08460	K00927	PGK	EC:2.7.2.3	phosphoglycerate kinase
NON14_08465	K00134	GAPDH	EC:1.2.1.12	glyceraldehyde 3-phosphate dehydrogenase
NON14_08835	K01835	pgm	EC:5.4.2.2	phosphoglucomutase
NON14_09225	K00016	LDH	EC:1.1.1.27	L-lactate dehydrogenase
NON14_09700	K04072	adhE	EC:1.2.1.10 1.1.1.1	acetaldehyde dehydrogenase / alcohol dehydrogenase

Table S3. Pyruvate metabolic pathway-associated genes involved in *Lb. sakei* HRB10

Locus tag	KO	Gene_name	EC	Definition
NON14_00995	K00925	ackA	EC:2.7.2.1	acetate kinase
NON14_01390	K23257	yvgN	EC:1.1.1.283 1.1.1.-	methylglyoxal/glyoxal reductase
NON14_01980	K00158	E1.2.3.3	EC:1.2.3.3	pyruvate oxidase
NON14_02830	K00925	ackA	EC:2.7.2.1	acetate kinase
NON14_03130	K00016	LDH	EC:1.1.1.27	L-lactate dehydrogenase
NON14_03875	K00626	E2.3.1.9	EC:2.3.1.9	acetyl-CoA C-acetyltransferase
NON14_04405	K01512	acyP	EC:3.6.1.7	acylphosphatase
NON14_04590	K00625	E2.3.1.8	EC:2.3.1.8	phosphate acetyltransferase
NON14_04820	K00925	ackA	EC:2.7.2.1	acetate kinase
NON14_05155	K20509	madB	EC:7.2.4.1	carboxybiotin decarboxylase
NON14_05190	K01571	oadA	EC:7.2.4.2	oxaloacetate decarboxylase (Na+ extruding) subunit alpha
NON14_05345	K00158	E1.2.3.3	EC:1.2.3.3	pyruvate oxidase
NON14_05575	K01006	ppdK	EC:2.7.9.1	pyruvate, orthophosphate dikinase
NON14_05855	K00161	PDHA	EC:1.2.4.1	pyruvate dehydrogenase E1 component alpha subunit
NON14_05860	K00162	PDHB	EC:1.2.4.1	pyruvate dehydrogenase E1 component beta subunit
NON14_05865	K00627	DLAT	EC:2.3.1.12	pyruvate dehydrogenase E2 component (dihydrolipoamide acetyltransferase)
NON14_05870	K00382	DLD	EC:1.8.1.4	dihydrolipoamide dehydrogenase
NON14_06115	K00873	PK	EC:2.7.1.40	pyruvate kinase
NON14_06395	K00656	E2.3.1.54	EC:2.3.1.54	formate C-acetyltransferase
NON14_07115	K01962	accA	EC:6.4.1.2 2.1.3.15	acetyl-CoA carboxylase carboxyl transferase subunit alpha
NON14_07120	K01963	accD	EC:6.4.1.2 2.1.3.15	/ acetyl-CoA carboxylase carboxyl transferase subunit beta
NON14_07125	K01961	accC	EC:6.4.1.2 6.3.4.14	acetyl-CoA carboxylase, biotin carboxylase subunit
NON14_07135	K02160	accB		acetyl-CoA carboxylase biotin carboxyl carrier protein
NON14_07305	K22373	larA	EC:5.1.2.1	lactate racemase
NON14_08445	K01759	GLO1	EC:4.4.1.5	lactoylglutathione lyase
NON14_09225	K00016	LDH	EC:1.1.1.27	L-lactate dehydrogenase
NON14_09240	K22212	mleA	EC:4.1.1.101	malolactic enzyme
NON14_09700	K04072	adhE	EC:1.2.1.10 1.1.1.1	acetaldehyde dehydrogenase / alcohol dehydrogenase

Table S4. ABC transporters-associated genes involved in *Lb. sakei* HRB10 genome.

Locus tag	KO	Gene_name	EC	Definition
NON14_00885	K19973	mntA	EC:7.2.2.5	manganese transport system ATP-binding protein
NON14_00890	K19976	mntB		manganese transport system permease protein
NON14_00895	K19975	mntC		manganese transport system substrate-binding protein
NON14_01010	K06726	rbsD	EC:5.4.99.62	D-ribose pyranase
NON14_01365	K18104	abcA	EC:7.6.2.2	ATP-binding cassette, subfamily B, bacterial AbcA/BmrA
NON14_01575	K18892	patB		ATP-binding cassette, subfamily B, multidrug efflux pump
NON14_01805	K05847	opuA	EC:7.6.2.9	osmoprotectant transport system ATP-binding protein
NON14_01810	K05845	opuC		osmoprotectant transport system substrate-binding protein
NON14_01940	K16785	ecfT		energy-coupling factor transport system permease protein
NON14_02290	K02040	pstS		phosphate transport system substrate-binding protein
NON14_02485	K16786	ecfA1	EC:7.-.-.-	energy-coupling factor transport system ATP-binding protein
NON14_02490	K16787	ecfA2	EC:7.-.-.-	energy-coupling factor transport system ATP-binding protein
NON14_02495	K16785	ecfT		energy-coupling factor transport system permease protein
NON14_02680	K02000	proV	EC:7.6.2.9	glycine betaine/proline transport system ATP-binding protein
NON14_02685	K02001	proW		glycine betaine/proline transport system permease protein
NON14_02690	K02002	proX		glycine betaine/proline transport system substrate-binding protein
NON14_03825	K10036	glnH		glutamine transport system substrate-binding protein
NON14_03830	K10038	glnQ	EC:7.4.2.1	glutamine transport system ATP-binding protein
NON14_04490	K11072	potA	EC:7.6.2.11	spermidine/putrescine transport system ATP-binding protein
NON14_04495	K11071	potB		spermidine/putrescine transport system permease protein
NON14_04500	K11070	potC		spermidine/putrescine transport system permease protein
NON14_04505	K11069	potD		spermidine/putrescine transport system substrate-binding protein
NON14_04950	K18889	mdlA		ATP-binding cassette, subfamily B, multidrug efflux pump
NON14_04955	K18890	mdlB		ATP-binding cassette, subfamily B, multidrug efflux pump
NON14_05700	K02071	metN		D-methionine transport system ATP-binding protein
NON14_05705	K02072	metI		D-methionine transport system permease protein
NON14_05710	K02073	metQ		D-methionine transport system substrate-binding protein
NON14_05835	K18887	efrA		ATP-binding cassette, subfamily B, multidrug efflux pump
NON14_05840	K18888	efrB		ATP-binding cassette, subfamily B, multidrug efflux pump
NON14_05995	K17077	artQ		arginine/lysine/histidine transport system permease protein
NON14_06000	K23060	artR	EC:7.4.2.1	arginine/lysine/histidine transport system ATP-binding protein
NON14_07100	K03523	bioY		biotin transport system substrate-specific component
NON14_07315	K02007	cbiM		cobalt/nickel transport system permease protein
NON14_07320	K02008	cbiQ		cobalt/nickel transport system permease protein
NON14_07325	K02006	cbiO		cobalt/nickel transport system ATP-binding protein
NON14_07720	K10823	oppF		oligopeptide transport system ATP-binding protein
NON14_07725	K15583	oppD		oligopeptide transport system ATP-binding protein
NON14_07730	K15582	oppC		oligopeptide transport system permease protein

NON14_07735	K15581	oppB		oligopeptide transport system permease protein
NON14_07740	K15580	oppA		oligopeptide transport system substrate-binding protein
NON14_08400	K05846	opuBD		osmoprotectant transport system permease protein
NON14_08405	K05845	opuC		osmoprotectant transport system substrate-binding protein
NON14_08410	K05846	opuBD		osmoprotectant transport system permease protein
NON14_08415	K05847	opuA	EC:7.6.2.9	osmoprotectant transport system ATP-binding protein
NON14_08630	K11051	ABC-2.CYL.B		multidrug/hemolysin transport system permease protein
NON14_08635	K11050	ABC-2.CYL.A		multidrug/hemolysin transport system ATP-binding protein
NON14_08655	K15580	oppA		oligopeptide transport system substrate-binding protein
NON14_08905	K02036	pstB	EC:7.3.2.1	phosphate transport system ATP-binding protein
NON14_08910	K02036	pstB	EC:7.3.2.1	phosphate transport system ATP-binding protein
NON14_08915	K02038	pstA		phosphate transport system permease protein
NON14_08920	K02037	pstC		phosphate transport system permease protein
NON14_08925	K02040	pstS		phosphate transport system substrate-binding protein
NON14_08945	K09811	ftsX		cell division transport system permease protein
NON14_08950	K09812	ftsE		cell division transport system ATP-binding protein

Table S5. Proteases and peptidases-associated genes involved in *Lb. sakei* HRB10

**Locus tag**	**KO**	**Gene_name**	**EC**	**Definition**
NON14_00490	K03100	lepB	EC:3.4.21.89	signal peptidase I
NON14_00540	K21471	cwlO	EC:3.4.-.-	peptidoglycan DL-endopeptidase CwlO
NON14_00660	K01259	pip	EC:3.4.11.5	proline iminopeptidase
NON14_00880	K08659	pepDA	EC:3.4.-.-	dipeptidase
NON14_00975	K21471	cwlO	EC:3.4.-.-	peptidoglycan DL-endopeptidase CwlO
NON14_00985	K08659	pepDA	EC:3.4.-.-	dipeptidase
NON14_01340	K01256	pepN	EC:3.4.11.2	aminopeptidase N
NON14_01410	K21471	cwlO	EC:3.4.-.-	peptidoglycan DL-endopeptidase CwlO
NON14_01610	K07258	dacC	EC:3.4.16.4	serine-type D-Ala-D-Ala carboxypeptidase (penicillin-binding protein 56)
NON14_01640	K08602	pepF	EC:3.4.24.-	oligoendopeptidase F
NON14_02315	K02654	pilD	EC:3.4.23.43 2.1.1.-	leader peptidase (prepilin peptidase) N-methyltransferase
NON14_02715	K01261	pepA	EC:3.4.11.7	glutamyl aminopeptidase
NON14_03600	K19689	ampS	EC:3.4.11.-	aminopeptidase
NON14_03635	K01265	map	EC:3.4.11.18	methionyl aminopeptidase
NON14_04115	K19220	cwlS	EC:3.4.-.-	peptidoglycan DL-endopeptidase CwlS
NON14_06295	K03100	lepB	EC:3.4.21.89	signal peptidase I
NON14_06340	K01419	hslV	EC:3.4.25.2	ATP-dependent HslUV protease, peptidase subunit HslV
NON14_06525	K03101	lspA	EC:3.4.23.36	signal peptidase II
NON14_06760	K08659	pepDA	EC:3.4.-.-	dipeptidase
NON14_06805	K01258	pepT	EC:3.4.11.4	tripeptide aminopeptidase
NON14_07330	K01297	ldcA	EC:3.4.17.13	muramoyltetrapeptide carboxypeptidase
NON14_08010	K21471	cwlO	EC:3.4.-.-	peptidoglycan DL-endopeptidase CwlO
NON14_08030	K01281	pepX	EC:3.4.14.11	X-Pro dipeptidyl-peptidase
NON14_08485	K08659	pepDA	EC:3.4.-.-	dipeptidase
NON14_09525	K01271	pepQ	EC:3.4.13.9	Xaa-Pro dipeptidase
NON14_09990	K08659	pepDA	EC:3.4.-.-	dipeptidase
NON14_10025	K21471	cwlO	EC:3.4.-.-	peptidoglycan DL-endopeptidase CwlO
NON14_10095	K08602	pepF	EC:3.4.24.-	oligoendopeptidase F
NON14_00420	K04771	degP	EC:3.4.21.107	serine protease Do
NON14_01110	K04086	clpL		ATP-dependent Clp protease ATP-binding subunit ClpL
NON14_02280	K03696	clpC		ATP-dependent Clp protease ATP-binding subunit ClpC
NON14_03175	K03798	ftsH	EC:3.4.24.-	cell division protease FtsH
NON14_03975	K03697	clpE		ATP-dependent Clp protease ATP-binding subunit ClpE
NON14_04650	K19225	gluP	EC:3.4.21.105	rhomboid protease GluP
NON14_05015	K11749	rseP	EC:3.4.24.-	regulator of sigma E protease
NON14_05920	K07177	K07177		Lon-like protease
NON14_05975	K03544	clpX		ATP-dependent Clp protease ATP-binding subunit ClpX
NON14_06075	K03695	clpB		ATP-dependent Clp protease ATP-binding subunit ClpB
NON14_06340	K01419	hslV	EC:3.4.25.2	ATP-dependent HslUV protease, peptidase subunit HslV
NON14_06345	K03667	hslU		ATP-dependent HslUV protease ATP-binding subunit HslU

NON14_08785	K01358	clpP	EC:3.4.21.92	ATP-dependent Clp protease, protease subunit

Table S6. Amino acid metabolism-associated genes involved in *Lb. sakei* HRB10

Locus tag	KO	Gene_name	EC	Definition
NON14_00330	K01939	purA	EC:6.3.4.4	adenylosuccinate synthase
NON14_00360	K10536	aguA	EC:3.5.3.12	agmatine deiminase
NON14_00365	K00926	arcC	EC:2.7.2.2	carbamate kinase
NON14_00370	K10536	aguA	EC:3.5.3.12	agmatine deiminase
NON14_00505	K01779	racD	EC:5.1.1.13	aspartate racemase
NON14_00510	K01834	PGAM	EC:5.4.2.11	2,3-bisphosphoglycerate-dependent phosphoglycerate mutase
NON14_00660	K01259	pip	EC:3.4.11.5	proline iminopeptidase
NON14_00845	K03781	katE	EC:1.11.1.6	catalase
NON14_01040	K01834	PGAM	EC:5.4.2.11	2,3-bisphosphoglycerate-dependent phosphoglycerate mutase
NON14_01320	K01439	dapE	EC:3.5.1.18	succinyl-diaminopimelate desuccinylase
NON14_01510	K01817	trpF	EC:5.3.1.24	phosphoribosylanthranilate isomerase
NON14_01520	K13954	yiaY	EC:1.1.1.1	alcohol dehydrogenase
NON14_01735	K09758	asdA	EC:4.1.1.12	aspartate 4-decarboxylase
NON14_02650	K13953	adhP	EC:1.1.1.1	alcohol dehydrogenase, propanol-preferring
NON14_02725	K01439	dapE	EC:3.5.1.18	succinyl-diaminopimelate desuccinylase
NON14_02730	K01439	dapE	EC:3.5.1.18	succinyl-diaminopimelate desuccinylase
NON14_02860	K04516	AROA1	EC:5.4.99.5	chorismate mutase
NON14_03080	K01929	murF	EC:6.3.2.10	UDP-N-acetylmuramoyl-tripeptide--D-alanyl-D-alanine ligase
NON14_03130	K00016	LDH	EC:1.1.1.27	L-lactate dehydrogenase
NON14_03250	K00286	proC	EC:1.5.1.2	pyrroline-5-carboxylate reductase
NON14_03440	K01756	purB	EC:4.3.2.2	adenylosuccinate lyase
NON14_03875	K00626	E2.3.1.9	EC:2.3.1.9	acetyl-CoA C-acetyltransferase
NON14_03885	K01641	E2.3.3.10	EC:2.3.3.10	hydroxymethylglutaryl-CoA synthase
NON14_04530	K00820	glmS	EC:2.6.1.16	glutamine---fructose-6-phosphate transaminase (isomerizing)
NON14_04700	K01915	glnA	EC:6.3.1.2	glutamine synthetase
NON14_05230	K01485;	codA	EC:3.5.4.1 3.5.4.21	cytosine/creatinine deaminase
NON14_05610	K00600	glyA	EC:2.1.2.1	glycine hydroxymethyltransferase
NON14_05695	K02437	gcvH		glycine cleavage system H protein
NON14_05870	K00382	DLD	EC:1.8.1.4	dihydrolipoamide dehydrogenase
NON14_06355	K01652	E2.2.1.6L	EC:2.2.1.6	acetolactate synthase I/II/III large subunit
NON14_06490	K01955	carB	EC:6.3.5.5	carbamoyl-phosphate synthase large subunit
NON14_06495	K01956	carA	EC:6.3.5.5	carbamoyl-phosphate synthase small subunit
NON14_06505	K00609	pyrB	EC:2.1.3.2	aspartate carbamoyltransferase catalytic subunit
NON14_06630	K00001	E1.1.1.1	EC:1.1.1.1	alcohol dehydrogenase
NON14_06770	K00891	aroK	EC:2.7.1.71	shikimate kinase
NON14_06775	K01735	aroB	EC:4.2.3.4	3-dehydroquinate synthase

NON14_06780	K03856	AROA2	EC:2.5.1.54	3-deoxy-7-phosphoheptulonate synthase
NON14_06785	K00014	aroE	EC:1.1.1.25	shikimate dehydrogenase
NON14_06800	K03785	aroD	EC:4.2.1.10	3-dehydroquinate dehydratase I
NON14_07035	K01919	gshA	EC:6.3.2.2	glutamate--cysteine ligase
NON14_07090	K08968	msrC	EC:1.8.4.14	L-methionine (R)-S-oxide reductase
NON14_07345	K01243	mtnN	EC:3.2.2.9	adenosylhomocysteine nucleosidase
NON14_07690	K00865	glxK	EC:2.7.1.165	glycerate 2-kinase
NON14_07950	K00764	purF	EC:2.4.2.14	amidophosphoribosyltransferase
NON14_07960	K23265	purQ	EC:6.3.5.3 3.5.1.2	phosphoribosylformylglycinamidine synthase subunit PurQ / glutaminase
NON14_07975	K01756	purB	EC:4.3.2.2	adenylosuccinate lyase
NON14_08315	K01953	asnB	EC:6.3.5.4	asparagine synthase (glutamine-hydrolysing)
NON14_08550	K01754	E4.3.1.19	EC:4.3.1.19	threonine dehydratase
NON14_08700	K00055	E1.1.1.90	EC:1.1.1.90	aryl-alcohol dehydrogenase
NON14_09080	K00789	metK	EC:2.5.1.6	S-adenosylmethionine synthetase
NON14_09145	K00020	HIBADH	EC:1.1.1.31	3-hydroxyisobutyrate dehydrogenase
NON14_09225	K00016	LDH	EC:1.1.1.27	L-lactate dehydrogenase
NON14_09700	K04072	adhE	EC:1.2.1.10 1.1.1.1	acetaldehyde dehydrogenase / alcohol dehydrogenase
NON14_09735	K00926	arcC	EC:2.7.2.2	carbamate kinase
NON14_09740	K00611	OTC	EC:2.1.3.3	ornithine carbamoyltransferase
NON14_09745	K01478	arcA	EC:3.5.3.6	arginine deiminase
NON14_09865	K01424	E3.5.1.1	EC:3.5.1.1	L-asparaginase
NON14_10005	K01752	E4.3.1.17	EC:4.3.1.17	L-serine dehydratase
NON14_10010	K01752	E4.3.1.17	EC:4.3.1.17	L-serine dehydratase

Table S7. Fatty acid biosynthesis pathway-associated genes involved in *Lb. sakei* HRB10

Locus tag	KO	Gene_name	EC	Definition
NON14_07110	K00208	fabI	EC:1.3.1.9 1.3.1.10	enoyl-acyl-carrier protein reductase I
NON14_07115	K01962	accA	EC:6.4.1.2 2.1.3.15	acetyl-CoA carboxylase carboxyl transferase subunit alpha
NON14_07120	K01963	accD	EC:6.4.1.2 2.1.3.15	acetyl-CoA carboxylase carboxyl transferase subunit beta
NON14_07125	K01961	accC	EC:6.4.1.2 6.3.4.14	acetyl-CoA carboxylase, biotin carboxylase subunit
NON14_07130	K02372	fabZ	EC:4.2.1.59	3-hydroxyacyl-acyl-carrier-protein dehydratase
NON14_07135	K02160	accB		acetyl-CoA carboxylase biotin carboxyl carrier protein
NON14_07140	K09458	fabF	EC:2.3.1.179	3-oxoacyl-acyl-carrier-protein synthase II
NON14_07145	K00059	fabG	EC:1.1.1.100	3-oxoacyl-acyl-carrier protein reductase
NON14_07150	K00645	fabD	EC:2.3.1.39	acyl-carrier-protein S-malonyltransferase
NON14_07160	K00648	fabH	EC:2.3.1.180	3-oxoacyl-acyl-carrier-protein synthase III
NON14_07165	K02372	fabZ	EC:4.2.1.59	3-hydroxyacyl-acyl-carrier-protein dehydratase
NON14_09010	K00059	fabG	EC:1.1.1.100	3-oxoacyl-acyl-carrier protein reductase
NON14_09175	K00059	fabG	EC:1.1.1.100	3-oxoacyl-acyl-carrier protein reductase
NON14_09870	K01071	MCH	EC:3.1.2.21	medium-chain acyl-acyl-carrier-protein hydrolase

Table S8. Fatty acid degradation pathway-associated genes involved in *Lb. sakei* HRB10

Locus tag	KO	Gene_name	EC	Definition
NON14_01520	K13954	yiaY	EC:1.1.1.1	alcohol dehydrogenase
NON14_02650	K13953	adhP	EC:1.1.1.1	alcohol dehydrogenase, propanol-preferring
NON14_03875	K00626	E2.3.1.9	EC:2.3.1.9	acetyl-CoA C-acetyltransferase
NON14_06630	K00001	E1.1.1.1	EC:1.1.1.1	alcohol dehydrogenase
NON14_09700	K04072	adhE	EC:1.2.1.10 1.1.1.1	acetaldehyde dehydrogenase / alcohol dehydrogenase
